# Mapping Functional Brain Activation Using [^14^C]-Iodoantipyrine in Male Serotonin Transporter Knockout Mice

**DOI:** 10.1371/journal.pone.0023869

**Published:** 2011-08-23

**Authors:** Raina D. Pang, Zhuo Wang, Lauren P. Klosinski, Yumei Guo, David H. Herman, Tansu Celikel, Hong Wei Dong, Daniel P. Holschneider

**Affiliations:** 1 Graduate Program in Neuroscience, University of Southern California, Los Angeles, California, United States of America; 2 Department of Psychiatry and Behavioral Science, University of Southern California, Los Angeles, California, United States of America; 3 Department of Neurology, University of Southern California, Los Angeles, California, United States of America; 4 Biomedical Engineering, University of Southern California, Los Angeles, California, United States of America; 5 Department of Cell and Neurobiology, University of Southern California, Los Angeles, California, United States of America; 6 Department of Neurology, School of Medicine, University of California Los Angeles, Los Angeles, California, United States of America; Mayo Clinic College of Medicine, United States of America

## Abstract

**Background:**

Serotonin transporter knockout mice have been a powerful tool in understanding the role played by the serotonin transporter in modulating physiological function and behavior. However, little work has examined brain function in this mouse model. We tested the hypothesis that male knockout mice show exaggerated limbic activation during exposure to an emotional stressor, similar to human subjects with genetically reduced transcription of the serotonin transporter.

**Methodology/Principal Findings:**

Functional brain mapping using [^14^C]-iodoantipyrine was performed during recall of a fear conditioned tone. Regional cerebral blood flow was analyzed by statistical parametric mapping from autoradiographs of the three-dimensionally reconstructed brains. During recall, knockout mice compared to wild-type mice showed increased freezing, increased regional cerebral blood flow of the amygdala, insula, and barrel field somatosensory cortex, decreased regional cerebral blood flow of the ventral hippocampus, and conditioning-dependent alterations in regional cerebral blood flow in the medial prefrontal cortex (prelimbic, infralimbic, and cingulate). Anxiety tests relying on sensorimotor exploration showed a small (open field) or paradoxical effect (marble burying) of loss of the serotonin transporter on anxiety behavior, which may reflect known abnormalities in the knockout animal's sensory system. Experiments evaluating whisker function showed that knockout mice displayed impaired whisker sensation in the spontaneous gap crossing task and appetitive gap cross training.

**Conclusions:**

This study is the first to demonstrate altered functional activation in the serotonin transporter knockout mice of critical nodes of the fear conditioning circuit. Alterations in whisker sensation and functional activation of barrel field somatosensory cortex extend earlier reports of barrel field abnormalities, which may confound behavioral measures relying on sensorimotor exploration.

## Introduction

In humans, a polymorphism in the serotonin transporter (5-HTT) promoter region (5-HTTLPR) affects the transcriptional efficiency of the transporter gene. Individuals carrying the low expressing form of the 5-HTTLPR polymorphism (the ‘s’ or ‘L_G_’ allele), which is associated with reduced transcription of 5-HTT and reduced serotonin (5-HT) uptake [1], [2], appear to have increased susceptibility to anxiety [1], [3] and mood symptoms in the face of environmental adversity [4] for review see [5], but not all studies find an effect [6], [7]. Because the effect of genes on behavior is often subtle, neuroimaging studies have provided new insight into the effects of the 5-HTTLPR polymorphism. Several neuroimaging studies have found that ‘s’ carriers of the 5-HTTLPR polymorphism display amygdala hyperactivation [8], [9], [10], which may be a result of abnormal functional connectivity between the prefrontal cortex (PFC) and the amygdala [11], [12].

5-HTT knockout mice (KO) offer a promising model for psychiatric research as parallels exist between the human polymorphism and the mouse model at the levels of serotonergic profile, behavior, physiological function, and stress hormone response [13], [14], [15], [16], [17] for review see [18]. Though 5-HTT KO animals lack high-affinity cellular uptake of 5-HT, 5-HT can be transported intracellularly with low efficiency (low affinity and selectivity) by the dopamine transporter [19] and polyspecific organic cation transporters [20], the latter of which have been shown to be upregulated in 5-HTT KO mice [20]. Thus, the 5-HTT KO mice have reduced, but not absent 5-HT clearance, an observation similar, though not analogous, to findings in the human 5-HTTLPR polymorphism.

The current study provides a detailed three-dimensional (3-D) map of functional brain activation during fear conditioned recall in the 5-HTT KO mouse, thereby exploring the possibility of reverse translation of brain functional responses in rodents. Specifically, we test the hypothesis that 5-HTT KO mice show an exaggerated limbic activation during a fear conditioned recall. Brain mapping is performed using an autoradiographic method [21], [22]. Perfusion autoradiography fills a gap in the current armamentarium of imaging tools in that it can deliver a 3-D assessment of functional activation of the awake, nonrestrained animal, with a temporal resolution of ∼5–10 seconds and a spatial resolution of 100 µm [23], [24]. This distinguishes it from other histological methods such as c-fos or cytochrome oxidase, which integrate brain responses over a duration of hours to days, or electrophysiological recordings, which typically only target very limited regions of the brain, or functional magnetic resonance imaging (fMRI) or positron emission tomography (microPET), which provide whole brain analysis, but require sedation of the animal. In addition, we perform experiments on whisker function to test the hypothesis that the exclusive use of anxiety tests reliant on sensory exploration cannot adequately access the anxiety phenotype in this model.

## Results

### Functional brain mapping during fear conditioned recall

#### Fear conditioning training and recall

**Figure 1:**
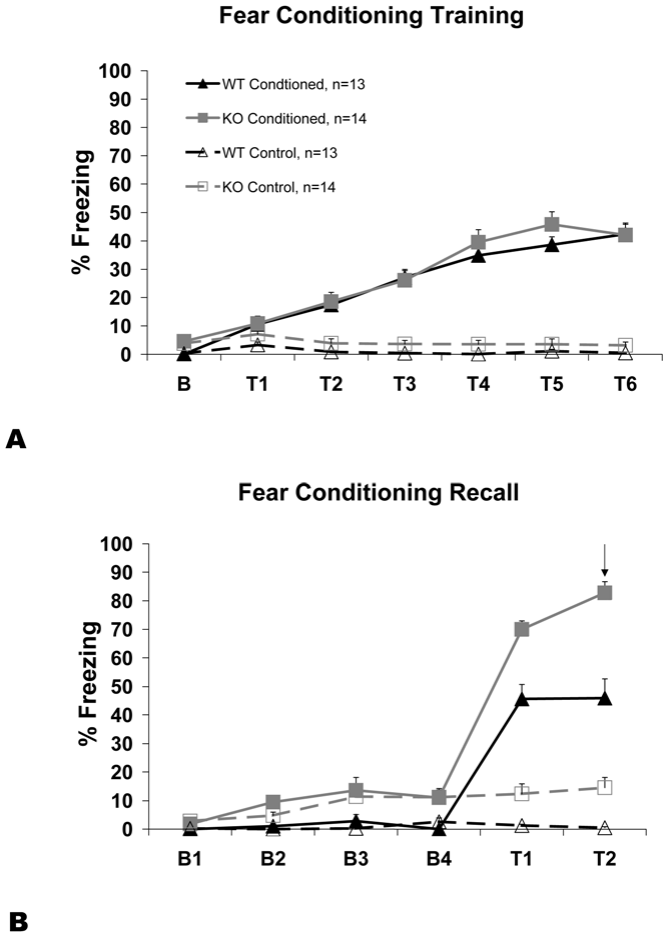
Fear Conditioning. 5-HTT KO mice exhibit increased freezing specific to recall in a fear conditioning paradigm A) Training: WT and KO animals show similar freezing levels during training of fear conditioning. Time on the x-axis is labeled as ‘B’ for the entire two-minute baseline and each ‘T’ represents the thirty second tone and the following one-minute quiet period. The y-axis represents percent time freezing during the specific time point. B) Recall: KO mice compared to WT mice show significantly increased freezing during baseline and recall of a tone previously associated with foot shock. Time on the x-axis is represented in 30-second bins with ‘B’ for baseline and ‘T’ for tone exposure. The y-axis represents percent time freezing during the specific time bin. The arrow indicates time of radiotracer injection for the imaging data. Error bars represent standard error of the mean (shown only unilaterally for graphical clarity).

During the training phase (day 1) conditioning significantly increased percent time freezing (conditioning: F_1, 49_ = 175.73; p<0.001) in a time dependent manner (time × conditioning: F_4, 195_ = 74.7; p<0.001). There was no significant genotypic difference in percent time freezing (genotype: F_1, 49_ = 2.63, p = 0.11), nor a significant interaction between genotype and conditioning (genotype × conditioning: F_1, 49_ = 0.08, p = 0.78; Figure 1a).

During recall testing (day 2), mice that were conditioned to the tone (CF) froze significantly more than control (CON) mice (conditioning: F_1, 49_ = 182.97, p<0.001) in a time dependent manner (time × conditioning: (F_1, 49_ = 198.81, p<0.001). 5-HTT KO mice froze significantly more than WT mice (genotype: F_1, 49_ = 51.39, p<0.001), with a significant interaction between genotype and conditioning (genotype × conditioning: F_1, 49_ = 5.7, p<0.001; Figure 1b). Genotypic differences in freezing response were significantly increased during tone exposure compared to the baseline condition (genotype × conditioning × time: F_1,49_ = 5.01, p = 0.03; Figure 1b).

#### Functional brain activation

During fear conditioning recall, the effects of conditioning and genotype on regional cerebral blood flow (rCBF) were assessed.

#### Factorial Analysis (Table 1, Figure 2)

Significant main effects of conditioning and of genotype were seen in several neocortical regions (frontal association cortex, FrA, primary, M1, and secondary, M2, motor cortex, primary, S1, including the barrel field, S1BF, and secondary, S2, somatosensory cortex), the amygdala (basolateral amygdala, BL, basomedial amygdala, BM, central amygdala, Ce, and lateral amygdala, La), the ventral hippocampus, the superior colliculi (SC), the raphe (dorsal, DR, and median, MnR), and midline cerebellum (Cb). In addition, a significant effect of conditioning was noted in the presubiculum (PrS), and a significant effect of genotype was noted in the cingulate cortex, insula, lateral orbital cortex (LO), retrosplenial cortex (RS), anterior amygdala area (AA), Postsubiculum (Post), parasubiculum (PaS), nucleus accumbens (Acb), caudate putamen (CPu, dorsal medial and ventral lateral), and inferior colliculus (IC). There was significant interaction between genotype and conditioning in the medial prefrontal cortex (prelimbic, infralimbic, and cingulate), medial orbital cortex (MO), M2, La, POST, PaS, Acb, CPu (dorsal medial and ventral lateral), midline thalamus, DR, and midline Cb.

**Figure 2:**
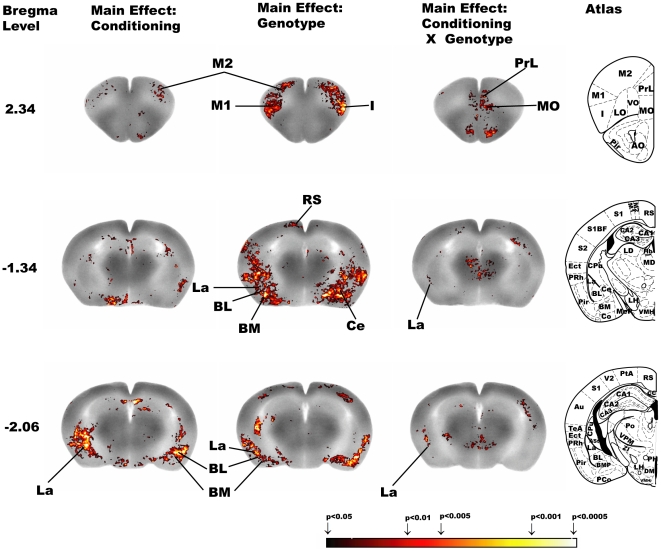
Factorial analysis examining the effect of genotype, conditioning or the interaction. Depicted are select coronal slices (anterior-posterior coordinates relative to bregma) of the template brain. Colored overlays show statistically significant effects of genotype or conditioning or their interaction, but do not reflect the direction of the effect. Abbreviations are from Franklin and Paxinos mouse atlas [67]: BL (basolateral amygdaloid nucleus), BM (basomedial amygdaloid nucleus), Ce (central amygdala), I (insular cortex), La (lateral amygdaloid nucleus), M1 (primary motor cortex), M2 (secondary motor cortex), MO (medial orbital cortex), PrL (prelimbic cortex), RS (retrosplenial cortex). Mouse brain atlas figures were reproduced from the mouse brain atlas [67] with modification and with permission from Elsevier.

**Table 1:** Significant changes in rCBF in the cortex and subcortex in the left and right hemispheres (L/R).

	WT	KO	CF	CON	Main Effect	Main Effect	
	CFvsCON	CFvsCON	KOvsWT	KOvsWT	CF	KO	CFxKO
**Cortex**							
Cingulate (Cg)	↓/-	-/-	-/-	↓/↓	-/-	#/#	#/-
Frontal association (FrA)	↓*/↓	↓*/↓	↑/↑	↑/↑	#/#	#/#	-/-
Infralimbic (IL)	↓/-	↑/↑	↑/↑	-/-	-/-	-/-	#/#
Insula (I)	-/↑*	-/-	↑*/↑	↑*/↑*	-/-	#/#	-/-
Motor: primary (M1)	↓/↓*	↓/↓	↑/↑	↑/↑	#/#	#/#	-/-
secondary (M2)	↓/↓*	↓*/↓	↑/↑	↑/↑	#/#	#/#	#/-
Orbital: lateral (LO)	↓/↓	↓/-	↑/↑	↑/↑	-/-	#/#	-/-
medial (MO)	↓/↓	↑/↑	↑/↑	-/-	-/-	-/-	#/#
ventral (VO)	↓/↓	-/-	↑/↑	-/-	-/-	-/-	-/-
Prelimbic (PrL)	↓/-	↑/↑	↑/↑	-/-	-/-	-/-	#/#
Retrosplenial (RS)	↑/-	-/-	↓*/↓	↓*/↓	-/-	#/#	-/-
Somatosensory: barrel field (S1BF)	-/↓	↓/-	↑/↑	↑/↑	#/-	#/#	-/-
primary (S1, non barrel field)	-/↓	↓/-	-/↑	↑/↑	#/#	#/#	-/-
secondary (S2, non barrel field)	-/↓	↓/↓	-/↑	↑/↑	#/#	#/#	-/-
**Subcortex**							
Accumbens nucleus (Acb)	↓*/↓	↑*/↑	↑/↑	↓/↓*	-/-	#/#	#/#
Amygdala:							
anterior amygdaloid area (AA)	-/-	-/-	-/↑	↑/↑	-/-	#/#	-/-
basolateral amygdaloid nucleus (BL)	↑*/↑	↑*/↑*	↑/↑	↑/↑	#/#	#/#	-/-
basomedial amygdaloid nucleus (BM)	↑*/↑	↑*/↑*	↑/↑	↑/↑	#/#	#/#	-/-
central amygdaloid nucleus (Ce)	-/-	↑*/↑*	↑/↑	↑/↑	#/-	#/#	-/-
lateral amygdaloid nucleus (La)	↑*/-	↑*/↑*	↑/↑	-/-	#/#	#/#	#/-
Cerebellum, midline (Cb)	↓	↑*	↑*	↓	#	#	#
Colliculi: inferior (IC)	↑*/-	-/-	↓*/↓*	↓/↓	-/-	#/#	-/-
superior (SC)	-/↓	↓*/↓	↓*/↓*	↓/↓	#/#	#/#	-/-
Hippocampus: ventral (vHPC)	↑/↑	↑/↑	↓*/↓*	↓/↓*	#/#	#/#	-/-
Raphe: dorsal, median (DR, MnR)	↓	-	-	↓	#	#	#
Striatum:							
dorsal medial caudate putamen (CPu)	↓/↓	-/-	↓/↓	↓/↓	-/-	#/#	#/#
ventral lateral CPu	-/↑	-/-	↑/↑	↑/↑	-/-	#/#	#/#
Subiculum: post (Post) & para (PaS)	-/-	↑*/↑	↓*/-	↓/↓*	-/-	#/#	#/#
presubiculum (PrS)	↑/↑	↑*/↑	-/↓	-/-	#/#	-/-	-/-
Thalamus: midline	↓	-	↑	↓	-	-	#

Arrows (↑**,** ↓) indicate the direction of rCBF change in the particular area and (-) indicates no significant change was noted. Areas significant after correction for multiple comparisons at the cluster level are marked with an *p<0.05. In addition, significance of the main effects of conditioning (CF) and genotype (KO), as well as their interaction on the ANOVA are noted as (#, p<0.05).

#### Effect of fear conditioning (WT: CF vs. CON and KO:CF vs. CON; Table 1, Figure 3)

Somatosensory and somatomotor cortex: Conditioned mice compared to controls in both genotypes showed a significant decrease in rCBF in M1, M2, S1, including S1BF, S2 somatosensory cortex, as well as in FrA. Medial prefrontal-orbitofrontal and insular cortex:

**Figure 3:**
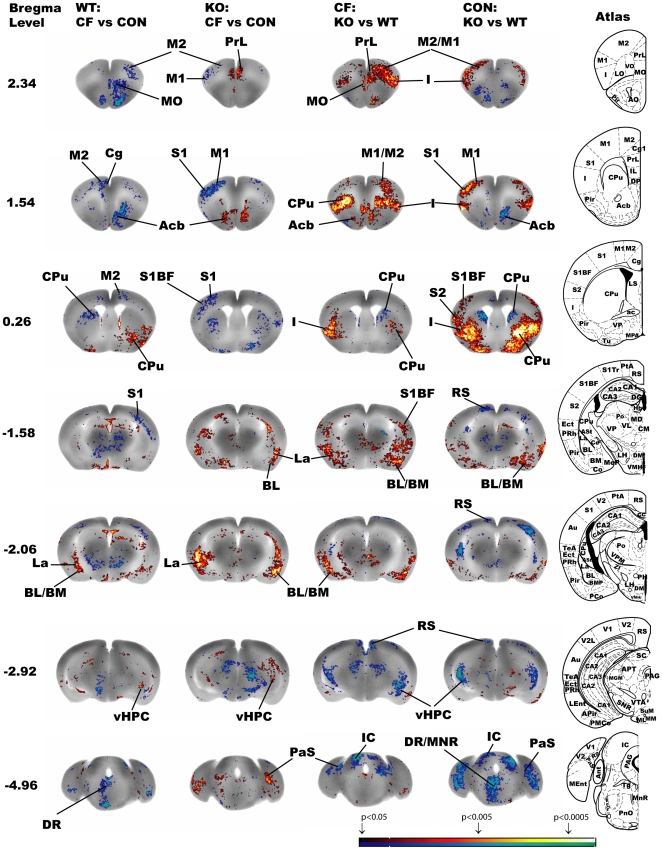
Changes in functional brain activity in mice in response to a fear conditioned tone. Depicted are select coronal slices (anterior-posterior coordinates relative to bregma) of the template brain. Colored overlays show statistically significant increased (red) and decreased (blue) differences for each comparison. Intragroup comparison were examined for fear conditioning with respect to the different genotypes (WT: CF vs. CON and KO: CF vs. CON) and genotype effects with respect to conditioning (CF: KO vs. WT and CON: KO vs. WT). Abbreviations are from Franklin and Paxinos mouse atlas [67]: Acb (accumbens nucleus), BL (basolateral amygdaloid nucleus), BM (basomedial amygdaloid nucleus), Cg (cingulate cortex), CPu (caudate putamen), DR (dorsal raphe nucleus), I (insular cortex), IC (inferior colliculus), La (lateral amygdaloid nucleus), M1 (primary motor cortex), M2 (secondary motor cortex), MnR (median raphe nucleus), MO (medial orbital cortex), PaS (parasubiculum), PrL (prelimbic cortex), RS (retrosplenial cortex), S1 (primary somatosensory cortex), S1BF (primary somatosensory, barrel field), S2 (secondary somatosensory cortex), ventral hippocampus (vHPC). Mouse brain atlas figures were reproduced from the mouse brain atlas [67] with modification and with permission from Elsevier.

In WT mice only, conditioned animals compared to controls showed significantly decreased rCBF in the medial prefrontal cortex (prelimbic, infralimbic, and cingulate). WT mice also showed significant decreases in the orbital cortex (MO, LO, and ventral, VO), and significant increases in insular and RS. In KO mice, conditioned mice compared to controls resulted in increased rCBF in the MO, IL and PrL and decreased rCBF in the LO. Amygdalar nuclei:

In both WT and KO mice, conditioning resulted in increased rCBF in the amygdala (BL, BM, and La). These results were confirmed after small volume correction of the amygdala (La, BL, BM), where conditioning significantly increased rCBF in conditioned compared to control animals in both genotypes (WT p<0.05, KO p<0.01). In KO mice only, conditioning also increased rCBF of the Ce. Hippocampal region: In both genotypes, conditioning resulted in increased rCBF of the ventral hippocampus and PrS. Post and PaS showed increased rCBF in KO mice only. Conditioning did not change rCBF in the dorsal hippocampus in either genotype. Cerebral nuclei: In WT mice only, conditioning increased rCBF in the ventral lateral CPu and decreased rCBF in the dorsal medial CPu, midline thalamus, and the Acb. In KO mice, conditioning resulted in increased rCBF in the Acb. Brainstem and cerebellum: Regardless of genotype, conditioning resulted in decreased rCBF of the SC. In WT mice only, conditioning increased rCBF in the IC, and decreased rCBF in the raphe (DR, and MnR). Conditioning resulted in differential activation patterns of the midline Cb (WT decrease rCBF, KO increase rCBF).

#### Effects of genotype (CF: KO vs. WT and CON: KO vs. WT; Table 1, Figure 3)

Somatosensory and somatomotor cortex: During recall, conditioned and control KO mice compared to their WT counterparts showed increased rCBF of the somatosensory and somatomotor cortical areas, including FrA, M1, M2, S1 (including S1BF), and S2. Medial prefrontal-orbitofrontal insular cortical areas: During recall KO compared to WT mice showed increased rCBF in the insular cortex and LO and decreased rCBF in the RS regardless of conditioning. In conditioned animals only, KO compared to WT mice showed increased rCBF of the infralimbic, prelimbic, MO, and VO. In control animals only, KO compared to WT mice showed a decreased rCBF in the cingulate. Amygdala and hippocampal region: KO mice in comparison to WT mice, regardless of conditioning status, showed increased rCBF in the amygdala (AA, BL, BM, Ce). In conditioned animals only, KO compared to WT mice showed increased rCBF in the La. After small volume correction, lack of 5-HTT significantly increased rCBF in the amygdala (p<0.01) in CF, but not in CON animals. KO compared to WT mice, regardless of conditioning status, showed decreased rCBF in the ventral hippocampus, Post and PaS. In conditioned animals only, KO compared to WT mice showed decreased rCBF in the PrS. There were no genotypic changes in the dorsal hippocampus for conditioned or control. Cerebral nuclei: KO compared to WT, regardless of conditioning status, resulted in increased rCBF in the ventral lateral CPu and decreased rCBF in the dorsal medial CPu. In both the Acb and midline thalamus, genotypic differences depended on the conditioning status (increase rCBF in CF, decrease rCBF in CON). Brainstem and cerebellum: KO compared to WT, regardless of conditioning status, resulted in decreased rCBF in the IC and SC. In control animals only, KO compared to WT resulted in decreased activation of the raphe (DR, MnR). In the midline Cb, genotypic differences depended on conditioning status (increased rCBF in CF, decreased rCBF in CON).

#### Correlation of functional activation with freezing scores (Table 2)

In CF animals of both genotypes, increased freezing scores were correlated with increased rCBF in the BL, the BM and the La and decreased rCBF in S1. In WT mice, increased freezing was also correlated with decreased rCBF in the M1, S2 and the dorsal hippocampus. In KO mice, increased freezing was correlated with decreased rCBF in the RS and increased rCBF in the dorsal hippocampus.

**Table 2:** Significant correlation of rCBF with behavioral freezing scores in the left and right hemispheres (L/R).

	CF: WT(L/R)	CF: KO(L/R)
**Cortex**		
Motor (M1)	-/↓	-/-
Retrosplenial (RS)	-/-	↓/↓
Somatosensory: primary (S1)	-/↓	↓/-
secondary (S2)	-/↓	-/-
**Subcortex**		
Amygdala:		
basolateral (BL)	↑**/-**	↑**/**↑*
basomedial (BM)	↑**/-**	↑**/**↑*
lateral (La)	↑**/-**	**-/**↑
Hippocampus: dorsal	↓**/**↓	↑^*^ **/-**

Arrows (↑, ↓) indicate a positive or negative correlation of rCBF with the behavioral freezing score. Areas significant after correction for multiple comparisons are marked with an *p<0.05.

### Anxiety tests reliant on sensorimotor exploration

#### Decreased exploration in 5-HTT KO mice in the novel open field

**Figure 4:**
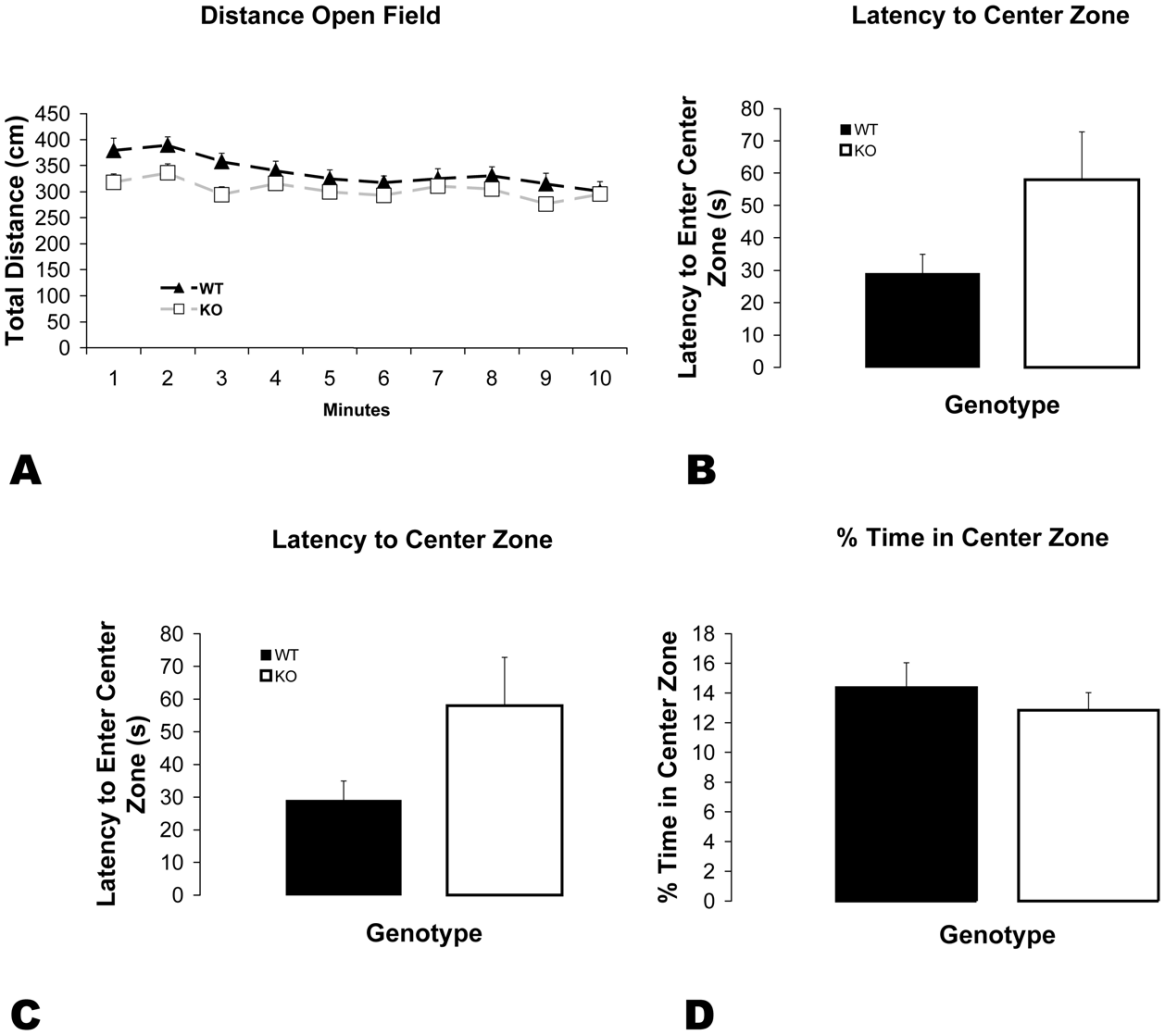
Open Field. A) 5-HTT KO mice show significantly decreased distance traveled throughout the arena in a novel open field. This is seen during the first 3 minutes of the test. B) KO mice show a non significant increase in latency to enter center zone C) There was no significant genotypic differences in number of entries into the center zone D) There was no significant genotypic difference in time spent in the center zone. Error bars represent standard error of the mean.

Locomotor activity in the open field decreased over time (time: F_5.7, 292.6_ = 4.95, p<0.001; Figure 4a). KO compared to WT mice showed significantly decreased exploratory locomotor activity in a novel open field (genotype: F_1, 51_ = 5.2, p<0.05; Figure 4a). Although there was an increase in latency to enter the center zone in the KO mice, this was not significant (p = 0.09; Figure 4b). There were no differences in other measurements traditionally used as a measure of anxiety: frequency of entry into center zone (p = 0.54; Figure 4c) and time in center zone (p = 0.46; Figure 4d).

#### Marble burying

**Figure 5:**
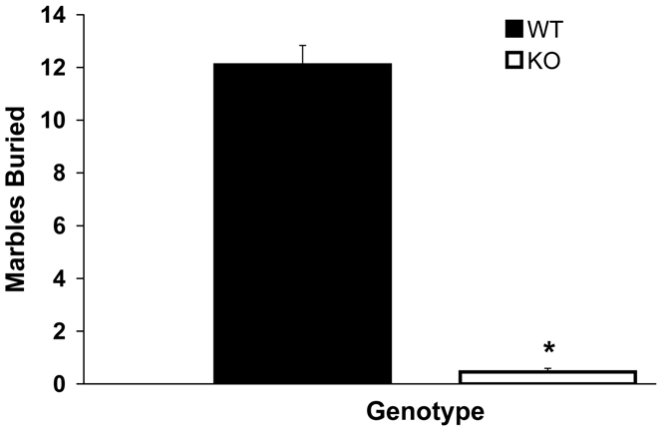
Marble Burying. 5-HTT KO mice compared to WT mice buried significantly less marbles placed in a novel cage; *p<0.001. Error bars represent standard error of the mean.

KO mice buried significantly fewer marbles than WT mice (Figure 5), which has been previously reported in 5-HTT KO mice [25], [26].

### Testing of whisker deficits

#### Impaired whisker function in 5-HTT KO mice

**Figure 6:**
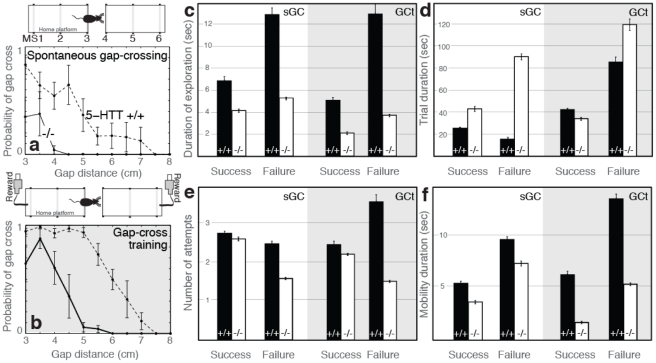
Impaired whisker function in 5-HTT KO mice. a) Top: Schematic view of the experimental set-up. Animals tried to locate a target object (i.e. platform) placed after a gap [64]. Their mobility is tracked using motion sensors. Bottom: Probability of successful object localization and subsequent gap-cross. 5-HTT deletion impairs tactile sensation (P<0.01, Kolmogorov-Smirnov test). b) Rewarded training for 3 weeks improves performance but does not rescue the whisker deficit. Top: Experimental set-up is similar to Figure 6a with the addition of computer controlled reward delivery ports. Bottom: Probability of gap-crossing (WT vs KO, P<0.01, Kolmogorov-Smirnov test). c) Duration of exploration, i.e. time spent at the gap, d) time animals required to travel between the two ends of the platform during successful trials or the delay to return to the starting position after sensory exploration at the gap during failures, e) number of attempts (i.e. visits to the gap) f) duration of mobility across genotypes and training conditions. Please refer to the text for statistical comparisons. Error terms are standard errors of the means.

5-HTT deletion impaired whisker sensation. On the spontaneous gap-crossing task (sGC), KO mice failed to locate the target object when the object was placed at whisker distances, 4.5–7.5 cm (Figure 6a). Moreover at shorter distances (3 cm < × <4.5 cm) KO animals failed significantly more often than WT mice (WT 0.66±0.13, KO 0.19±0.11, values are mean ± SEM, p<0.01). This impairment was not due to lack of sensory exploration (Figure 6c) although KO mice explored the gap for shorter periods than WT mice independent from whether they ultimately located the target (p<0.01) or failed to do so (p<0.001). Longer duration of sensory exploration in those trials that KO mice failed to locate the target, compared to successful trials, suggest that duration of exploration was not the cause of the failures. The sensory deficit was not due to lack of motivation as KO mice spent more time performing the task (Figure 6d; all trials combined, WT 20.2±1.0 seconds, n: 2674, KO 79.0±2.2, n: 2036, p<0.001) and made as many attempts, i.e. visits to the gap in a trial, to locate the target (Figure 6e; All trials combined, WT 2.5±0.004, KO 1.8±0.03, p>0.05). Number of attempts required to locate the target in successful trials did not differ across the genotypes (p>0.05), although during failures KO animals visited the gap significantly less often than WT mice (Figure 6e, p<0.05). Although the sensory deficit was not a reflection of a general lack of motor activity on the task (see above), mobility of the KO mice was significantly less than WT mice (Figure 6f; all trials combined, WT 7.7±0.2, KO 6.6±0.2, p<0.05).

#### Repeated rewarded training did not rescue the sensory deficit in 5-HTT KOs

The sensory deficit in KO mice persisted even when the animals were rewarded for gap-crossing and trained on the task for 3 continuous weeks (Figure 6c). This appetitive gap-cross training (GCt) increased the likelihood, for both WT and KO animals, to locate the target object when it was placed at whisker distances (WT: sGC 0.15±0.09, GCt 0.37±0.19, p<0.05; KO: sGC 0±0, GCt 0.02±0.01) or closer (WT: sGC 0.68±0.12, GCt 0.96±0.02, p<0.05; KO: sGC 0.19±0.10, GCt 0.62±0.11, p<0.01). Although KO animals crossed significantly larger distances during GCt compared to sGC (Figure 6a, b), their performance in the whisker distances was still impaired (p<0.01). As in the case of sGC, KO mice explored the gap for shorter periods than the WT mice (Figure 6c; p<0.01) and had reduced number of visits to the gap, attempting to find the target (Figure 6e; p<0.05). Furthermore KO mice were less mobile on the task (Figure 6f; p<0.01). Increased duration of mobility and sensory exploration during failures, compared to successful trials, argue that the sensory deficit observed in KO mice is not due to lack of sensory exploration, motivation or a generalized motor deficit. Accordingly time it took for the KO mice to complete successful trials were largely comparable to the WT mice (Figure 6d; Successful trials: WT 41.8±1.4, KO 33.3±1.3, p>0.05; Failures: WT 84.6±4.6, KO 118.4±5.1, p>0.05).

## Discussion

During recall of a previously conditioned tone, 5-HTT KO mice in comparison to WT mice showed increased anxiety behavior (freezing), increased rCBF in the amygdala, insula, and barrel field cortex, decreased rCBF in the ventral hippocampus, and conditioning dependent rCBF changes in the medial prefrontal (mPFC) regions (prelimbic, infralimbic, and cingulate). Anxiety tests relying on sensorimotor exploration of the environment reproduced less clearly the anxious phenotype of the KO mice shown in the conditioned fear paradigm. These latter findings are consistent with the impaired whisker sensation of the KO mice in the spontaneous and appetitive gap crossing tasks.

### Functional brain mapping during fear conditioned recall

During training, mice that were being conditioned to the tone showed progressively increased freezing behavior during training. Genotype did not have a significant effect on freezing behavior during the training phase. This finding was noted previously by Wellman et al [16] and is consistent with the reported absence of genotypic differences in footshock sensitivity [17]. During recall, mice that were conditioned to the tone showed significantly increased freezing behavior to the tone. KO compared to WT mice displayed increased freezing in the conditioned and the control (no-footshock) groups, suggesting that KO animals were sufficiently ‘sensitized’ to allow the tone by itself to elicit a partial fear response.

The amygdala is believed to play an important role in conditioned auditory perception [27]. The lateral amygdala (La) relays information to the central amygdala (Ce), whose efferents are critical to eliciting the behavioral, neurohumeral, and sympathetic responses characterizing states of fear [27]. Functional brain mapping during fear conditioned recall showed a main effect of conditioning in the amygdala (La, BL, BM). Conditioned animals compared to controls showed greater activation of the amygdala, with amygdalar activation correlating positively with freezing scores in both KO and WT mice. Genotype itself showed a main effect in the amygdala (La, BL, BM, Ce), with KO mice compared to WT mice showing increased amygdala activation (KO > WT). There was an interaction between genotype and conditioning in the La. Even in control mice, amygdala (BL, BM, Ce) activation was greater in KO than WT mice, which is consistent with their increased freezing behavior during fear conditioned recall.

The mPFC, via modulation of amygdalar activation, is thought to be necessary for the normal expression and extinction of conditioned fear [28], [29]. During recall, there was a genotype × conditioning interaction in activation of the ventral mPFC (prelimbic and infralimbic), with the most significant changes in activation in KO animals exposed to the footshock (KO-CF > KO-CON, WT-CF). Because prelimbic and infralimbic activity are thought to have opposing roles in the modulation of fear responses [30], the exact interpretation of these results is unclear, but suggest that 5-HTT deficits result in exaggerated activity of both regions to stress. Its relationship to reported morphological abnormalities in KO mice in this region remains to be clarified [16].

The anterior cingulate has been shown to modulate the efficiency of fear related learning [29], while the role of the retrosplenial (RS, posterior cingulate) in auditory fear conditioning remains unresolved [31], [32]. During recall, there was a genotype × conditioning interaction in activation of the cingulate, with the most significant changes in activation in control KO compared to WT mice (KO-CON > WT-CON). KO controls compared to WT controls showed deactivation of the cingulate and RS alongside activation of the amygdala, and greater fear-related behavioral immobility. This pattern of activation is consistent with the concept of a decrease in cortical inhibitory effects on the amygdala [33]. Fear conditioned KO compared to KO controls or fear conditioned WT mice demonstrated no changes in the cingulate, possibly due to a floor effect.

The insular cortex, which strongly innervates the amygdala, is thought to play an integral role in providing the amygdala with aversive sensory information [34], as well as in integration of autonomic responses [35]. Our results showed that genotype, rather than conditioning, was the dominant determinant of insular activation (KO-CF, KO-CON > WT-CF > WT-CON), which was confirmed by the factorial analysis. Hyperactivation of the insula in KO mice is consistent with the idea that these mice may focus more on aversive sensory signals that carry emotional significance.

There is a general consensus that the ventral hippocampus plays a role in the acquisition of tone conditioning [36], [37], [38], whereas the dorsal hippocampus is required for contextual cues and spatial navigation [38], [39]. Regardless of genotype, conditioning resulted in increased activation in ventral, but not dorsal, regions of the hippocampus. KO compared to WT mice displayed decreased activation of the ventral hippocampus regardless of conditioning. The interpretation of this remains unclear, but may reflect serotonergic effects on the morphofunctional development of the hippocampus [40] and synaptic plasticity in the hippocampus [41], [42].

Conditioning reduced activity in the motor cortex (M1, M2) and somatosensory cortex (S1, S1BF, S2). Freezing scores in tone-conditioned animals were negatively correlated with activity in M1 and S2 of WT animals, and S1 in both genotypes, consistent with increased motor immobility and possibly decreased somatosensory processing in animals with the highest freezing. Additionally, our results demonstrate increased functional activation of the S1BF in KO compared to WT animals, which may reflect an attempt to overcompensate for abnormalities in the KO's anatomy and electrophysiological function in the barrel field cortex [43], [44].

5-HTT has been implicated in playing a role in blood pressure and vascular reactivity [45]. A recent study has shown that awake, nonanesthetized 5-HTT KO rats show no alterations in diurnal mean arterial pressure and heart rates [46]. This likely is due to the fact that lifelong abnormalities in 5-HTT results in compensatory mechanisms in the vascular system. Acute application of 5-HT to the aorta in-vitro elicited greater contractions in KO than in WT animals [46], suggesting that acute vs. chronic alterations in KO rats results in different effects. Small changes in blood pressure, if they do occur, would likely have little effect on changing CBF because of autoregulation. However, we cannot rule out that, in theory, differences in rCBF might be a result of general effects of 5-HT on the regulation of blood flow, rather than specific effect of the paradigm.

### Anxiety tests reliant on sensorimotor exploration

Anxiety tests reliant on whisker exploration (e.g. open field, marble burying) may not adequately assess the anxious phenotype in the 5-HTT KO mouse model, because of the known abnormalities in the barrel field cortex of KO mice which maps the whiskers [43], [44]. These abnormalities, however, are unlikely to directly affect behaviors that are relatively independent of whisker function (e.g. fear conditioning).

Indeed, in the open field, an anxiety test reliant on sensorimotor exploration, KO compared to WT mice showed only a small increase in anxiety-like responses (i.e. reduced exploratory behavior). Marble burying, a test of ‘defensive behavior’ [47] that is reliant on burrowing [48], in fact showed a ‘paradoxical’ (nonanxious) response in the KOs, a result which has been previously reported [25], [26]. However, anxiety in the KO mouse was robustly represented during fear conditioned recall.

### Testing of whisker deficits

To further explore the behavioral effects of documented abnormalities in the somatosensory cortex of 5-HTT KO mice, an additional group of experimentally naïve male mice were tested on a learning task dependent on intact whisker function. In this task, the mouse was placed on one of two platforms with a variable gap-distance between the platforms. In the presence of white noise and darkness, at distances where the mouse could not easily touch with the paw or nose, the mouse had to rely on its whiskers to successfully localize and cross to the opposing platform. Thus, this task allowed for quantification of unrestrained whisker-based tactile exploration. This study confirmed impaired whisker sensation in 5-HTT KO mice, a result which extends earlier anatomic and electrophysiologic reports of abnormalities in the somatosensory system [43], [44] for review [49].

The effects of the 5-HTT gene knockout on other somatosensory systems (tactile, etc.) is an area of active investigation. Relevant to this study, evaluation of footshock sensitivity has revealed no genotypic differences [17]. This is consistent with our observation and that of others [16] of no genotypic differences in freezing behavior during the training phase where mice received acute footshocks. This suggests that the KO animals are able to adequately respond to the incoming footshock-related somatosensory information; thus the increased freezing responses noted in KO compared to WT mice during recall are not mediated by altered perception in the mouse footpad. In any case, prior reports of hypoalgesic responses to noxious stimulation in other sensory modalities (visceral, temperature, mechanical, inflammatory) [50], [51], [52] would be predicted to result in lesser, rather than the increased fear responses seen during recall.

### Conclusions

Recently there has been concern about the predictive validity of current animal models of behavioral disorders [53]. Emphasis has been placed on going beyond behavioral endpoints and deconstructing psychiatric symptom - based syndromes into biological endophenotypes [54], [55]. The purpose of such endophenotypic ‘biomarkers’ is to divide behavioral symptoms into more stable phenotypes with a clear genetic connection. Functional brain mapping has been proposed as such an endophenotype. The increased functional activation of the amygdala and altered patterns of activation in the mPFC (infralimbic, prelimbic, cingulate) of KO mice shown in this study parallels neuroimaging findings in humans that are carriers of the low expressing form of 5-HTTLPR [8], [9], [10], [11], [12]. While KOs do not fully reproduce the human 5-HTTLPR polymorphism, they share the common biological effect of diminished (but not wholly absent) 5-HT reuptake.

By providing a detailed 3-D map of functional brain activity in the mouse involved in the regulation of emotional function, this study provides evidence of the translation of human neuroimaging studies to the animal model. This type of endophenotypic measurement is essential for further understanding the validity of the 5-HTT KO animal model, in which sensory deficits may confound results from anxiety tests reliant on sensorimotor exploration. Furthermore, this study extends our understanding of the effects of 5-HTT on modulating central processing in several brain regions, which could provide the basis for future directed molecular studies evaluating the effect of 5-HTT on neural substrates.

## Methods

### Ethics Statement

All experimental protocols were approved by the Institutional Animal Care and Use Committee of the University of Southern California (Animal Welfare Assurance # A3518-01, USC protocol # 11093).

### Animals

Mice were bred at the university vivarium from pairs obtained from Taconic (Taconic, Hudson, NY). Mice had been backcrossed onto a C57BL/6 background for greater than 15 generations from an original mixed background [129/P1ReJ (ES cells), C57BL/6J and CD-1] [56], [57]. Male mice were weaned at 3 weeks, housed in groups of 3–4 on a 12 hour light/dark cycle (lights on at 0600) until 3 months of age with direct contact bedding and free access to rodent chow (NIH #31M diet) and water. At the start of behavioral testing, animals were individually housed. Genotyping was performed by Transnetyx, Inc. (Cordova, TN) from tail snips obtained post mortem with primer sequences obtained from Taconic (m5-HTT-C: 5′ TGA ATT CTC AGA AAG TGC TGT C 3′, m5-HTT-D: 5′ CTT TTT GCT GAC TGG AGT ACA G 3′, neo3a: 5′ CAG CGC ATC GCC TTC TAT C 3′). All behavioral testing was conducted during the light phase of the light/dark cycle (0930 to 1430).

### Functional brain mapping during fear conditioned recall

#### Surgery

Surgery was initiated one week after Open Field testing (described below). Animals were anesthetized with isoflurane (2.0%). The ventral skin of the neck was aseptically prepared and the right external jugular vein was catheterized with a 1-French silastic catheter (SAI infusion, Chicago, IL), which was advanced 1 cm into the superior vena cava. The catheter was externalized through subcutaneous space to a dorsal percutaneous port. The catheter was filled with 0.01 mL Taurolidine-Citrate lock solution (SAI infusion, Chicago, IL) and was closed with a stainless steel plug (SAI infusion, Chicago, IL).

#### Conditioned fear- training phase

Fear conditioning experiments [58] were conducted at three days post surgery. Animals were habituated to the experimental room for thirty minutes in the home cage. Thereafter, mice were placed in a Plexiglas box (22.5 cm ×21 cm ×18 cm) with a floor of stainless steel rods. The chamber was illuminated with indirect ambient fluorescent light from a ceiling panel (930 lx) and was subjected to background ambient sound (65 dB). After a two minute baseline, the animals were presented a tone six times (30 s, 70 dB, 1000 Hz/8000 Hz continuous, alternating sequence of 250 ms pulses). Each tone was separated by a one minute quiet period. In the conditioned fear (CF) groups (KO-CF: body weight  = 27 g±0.6 g, age = 12.4 wks±0.3 wks, n = 12; WT-CF: body weight =  26 g±0.5 g, age  = 12.8 wks±0.3 wks, n = 13) each tone was immediately followed by a foot shock (0.5 mA, 1 s). Control (CON) animals (KO-CON: body weight =  27 g±0.4 g, age = 12.4 wks±0.2 wks, n = 13; WT-CON: body weight =  26 g±0.3 g, age = 12.4 wks ±0.2 wks, n = 11) received identical exposure to the tone but without the foot shock. One minute following the final tone, mice were returned to their home cages.

#### Functional neuroimaging during conditioned fear recall

Twenty-four hours after the training session, animals were placed in the experimental room for one hour in their home cage. Thereafter, the animal's percutaneous cannula was connected to a tethered catheter containing the perfusion radiotracer ([^14^C]-iodoantipyrine, 325 µCi/kg in 0.180 mL of 0.9% saline, American Radiolabelled Chemicals, St. Louis, MO) and a syringe containing a euthanasia solution (50 mg/kg pentobarbital, 3 M KCl). Animals were allowed to rest in a transit cage for ten minutes prior to exposure to the behavioral cage (a cylindrical, dimly lit (300 lx), Plexiglas cage with a flat, Plexiglas floor). CF and CON animals received a two minute exposure to the behavioral cage context followed by a one minute continuous exposure to the conditioned tone. One minute after the start of the tone exposure, the radiotracer was injected intravenously at 1.0 mL/min using a mechanical infusion pump (Harvard Apparatus, Holliston, MA), followed immediately by injection of the euthanasia solution. This resulted in cardiac arrest within 5–10 seconds, a precipitous fall of arterial blood pressure, termination of brain perfusion, and death. Brains were rapidly removed and flash frozen in methylbutane/dry ice.

#### Behavioral analysis of conditioned fear

Behaviors were recorded using Windows Movie Makes (Microsoft) by a camera placed in front of the cage. The duration of the animal's freezing response, defined as the absence of all visible movements of the body and vibrissae aside from respiratory movement, served as the behavioral measure of conditioned fear memory. Behaviors were analyzed in a blinded fashion using the Observer 8.0 (Noldus Inc., Leesburg, VA). The freezing data were transformed to a percentage of time spent freezing. Statistical comparison was performed with a repeated measure analysis of variance (ANOVA) using “genotype” and “conditioning” as between subject factors. The repeated measure was “time” (time intervals during training were 90 s, i.e. 30 s tone followed by a 1 minute quiet period, time intervals during recall were baseline and tone).

#### Autoradiography

Brains were sliced in a cryostat at −20°C in 20 µm sections, with an interslice spacing of 140 µm. Slices were heat dried on glass slides and exposed to Kodak Ektascan diagnostic film (Eastman Kodak, Rochester, NY USA) for 14 days at room temperature along with twelve [^14^C] standards (Amersham Biosciences, Piscataway, NJ). Autoradiographs were then digitized on an 8-bit gray scale using a voltage stabilized light box (Northern Lights Illuminator, InterFocus Ltd., England) and a Retiga 4000R charge-coupled device monochrome camera (Qimaging, Canada). Cerebral blood flow (CBF) related tissue radioactivity was measured by the classic [^14^C]-iodoantipyrine method [21], [22]. In this method, there is a strict linear proportionality between tissue radioactivity and CBF when the data is captured within a brief interval (∼10 seconds) after the tracer injection [59], [60].

#### 3-D reconstruction of the digitized autoradiographs

3-D reconstruction has been described in our prior work [61]. In short, regional CBF (rCBF) was analyzed on a whole-brain basis using statistical parametric mapping (SPM, version SPM5, Wellcome Centre for Neuroimaging, University College London, London, UK). SPM, a software package was developed for analysis of imaging data in humans [62], has recently been adapted by us and others for use in brain autoradiographs [61], [63], [64]. A 3-D reconstruction of each animal's brain was conducted using 69 serial coronal sections (starting at slice bregma 2.98 mm) and a voxel size of 40 µm ×140 µm ×40 µm. Adjacent sections were aligned both manually and using TurboReg, an automated pixel-based registration algorithm [65]. After 3-D reconstruction, all brains were smoothed with a Gaussian kernel (FWHM  = 120 µm ×420 µm ×120 µm). The smoothed brains from all groups were then spatially normalized to the smoothed reference brain (one “artifact free” brain). Following spatial normalization, normalized images were averaged to create a mean image, which was then smoothed to create the smoothed template. Each smoothed original 3-D reconstructed brain was then spatially normalized into the standard space defined by the smoothed template [61].

#### SPM

An unbiased, voxel-by-voxel analysis of whole-brain activation using SPM was used for detection of significant changes in functional brain activation. Voxels for each brain failing to reach a specified threshold (80% of the mean voxel value) were masked out to eliminate the background and ventricular spaces without masking gray or white matter. Global differences in the absolute amount of radiotracer delivered to the brain were adjusted in SPM for each animal by scaling the voxel intensities so that the mean intensity for each brain was the same (proportional scaling). Using SPM, a factorial ANOVA was implemented at each voxel testing the null hypothesis that there was no genotypic or fear conditioning effect, as well as the interaction between genotype and conditioning (F_1, 44_, p<0.05). After running the factorial analysis, we implemented a Student's t-test (unpaired) at each voxel to determine directionality of significance. Significance (p<0.05) was established at the cluster level (minimum cluster extent of 100 contiguous voxels) with and without a correction for multiple comparisons. Brain regions were identified using coronal, sagittal and transverse views from the mouse brain atlas [66], [67]. To increase power in the amygdala (combined La, BL, and BM) a small volume correction was also performed. ROIs of the amygdala (bilateral, combined La, BL, and BM), using 10 serial slices starting at bregma −0.94) were manually drawn on the template brain and a small volume correction was performed for each of the comparisons (WT: CF vs. CON, KO: CF vs. CON, CF: KO vs. WT, CON: KO vs. WT). Significance was set for p<0.05 after correction for multiple comparisons by the SPM software. To detect brain regions showing rCBF correlated with fear responses, SPM analysis using freezing score as an individual covariate was run for the CF mice of both genotypes. Significance level was set at p<0.05 for Pearson's correlation coefficient.

### Anxiety tests reliant on sensorimotor exploration

#### Open field

Mice (KO n = 28, WT n = 25) were habituated for 30 minutes to the behavioral room. They were then placed in the bottom portion of a test chamber (a novel circular arena, diameter 42.5 cm, height 11.5 cm), which was illuminated from the ambient fluorescent light from the ceiling (558 lx), and allowed to freely explore for 10 minutes. Latency to enter the center zone (diameter 16.5 cm), time spent in the center zone, and frequency of entries into the center zone was assessed for each animal from the digitized video recordings using EthoVision 3.1 (Noldus, Inc., Leesburg, VA). Group averages were compared using a t-test (two tailed, p<0.05). Path length traveled in each one minute interval in the arena was calculated for each animal. A repeated ANOVA was performed on path length using “genotype” as a between subject factor and “time” as a within subject factor.

#### Marble burying

A separate group of male mice (n = 11/group) were tested in a marble burying paradigm [68]. Each mouse was placed in a novel cage filled with one inch of cozy critter super shavin's bedding (International Absorbants Inc., Ferndale, WA). Twenty-five small blue glass marbles (10–12 mm diameter) were clustered in the center of the cage. Mice were placed in the front of the cage facing the marbles and allowed to explore for thirty minutes. Thereafter mice were returned to their home cage and the number of marbles buried (>2/3 of the marble buried with bedding) was counted. Group averages of marbles buried were compared using a t-test (two tailed, p<0.05).

### Testing of whisker deficits

#### Spontaneous gap crossing (sGC)

The apparatus and training procedures have been described before [69]. In short, after initial habituation to the experimenter and the apparatus, individual animals (n = 4/group) were placed on one of the two elevated platforms separated from each other with randomly varying gap-distance (range: 3–8 cm, step-size: 0.5 cm) and their probability of successful object localization across gap-distances was quantified. The training was performed under infrared light and white noise; the platforms were cleaned using 70% isopropanol between sessions. Animal mobility on the platforms was quantified using custom-made infrared motion sensors placed at the two ends and the middle of each platform. Trial duration, duration of sensory exploration at the gap, number of attempts prior to successful gap-crossing, and duration of mobility were quantified and genotypes were compared using Student's t-test. Animals had *ad libitum* access to the food and water at all times, except when they were performing the task (1 session/day for 7 days; session duration: 30 min). Animals were not baited for successful task execution.

#### Gap-Cross training (GCt)

GCt [70] was similar to the sGC with the exception that the animals were food deprived (to ∼90% of their free-feeding rate) throughout the training period and were rewarded (1 pellet, 14 mg/pellet, BioServ, product #F05684) for successful gap crossing on the task. Unlike in the sGC, with repeated GCt animals increase their probability of successful object localization. The training apparatus and quantification of the variables were as described above. Each animal (n = 4/group) received 3 weeks of training on the apparatus (1 session/day; 7 sessions/week; session duration: 30 min). Tactile exploration of the animal onto the target platform was recorded using a high-speed camera (Allied Vision Technologies, Model: Pike) at 300 fps and a human observer confirmed that animals performed the task using their whiskers.
